# Validation of circular dichroic spectroscopy of synthetic oligonucleotide PS2.M for ${\hbox {K}}^{+}$ concentration measurements

**DOI:** 10.1140/epjp/s13360-022-02581-2

**Published:** 2022-04-07

**Authors:** Luca Bruni, Massimo Manghi, Paola De Sanctis, Cinzia Zucchini, Simonetta Croci

**Affiliations:** 1 National Institute for Biostructures and Biosystems, Rome, Italy; 2 Department of Medicine and Surgery, University of Parma, Parma, Italy; 3 Department of Experimental, Diagnostic and Specialty Medicine (DIMES), Unit of Histology, Embryology and Applied Biology, Università di Bologna, Bologna, Italy

## Abstract

The single-stranded synthetic oligonucleotide PS2.M is known to provide a basis for developing sensors since it tends to fold into structures called G-quadruplexes (G4) having characteristic topology and orientation with probabilities that depend on the chemical environment. The presence and concentration of cation species are among the key factors that determine the outcome of such a process. PS2.M and other aptamers have been used in several applications in conjunction with various probes, such as hemin, at the cost of increased technical complexity and applicability limitations. We instead validated the application limits of Circular Dichroic spectroscopy (CD) as only measurement method to assay PS2.M as ${\hbox {K}}^{+}$ sensor in a variety of solutions having different chemical complexity. The tested solutions range from simple $\hbox {NaCl}$ and $\hbox {KCl}$ solutions to chemically complex solutions like DMEM—Dulbecco’s Modified Eagle Medium—which is widely used in a biological laboratory. PS2.M was also evaluated in solutions of ${\hbox {KHCO}}_{3}$and D-ribose (K:D-rib), an antioxidant potassium compound, to compare its response to the simple $\hbox {KCl}$ solution case. Our findings show that, within specific concentration applicability ranges, CD spectra can estimate the ${\hbox {K}}^{+}$ concentration in the examined water solutions even at high ${\hbox {Na}}{+}$ concentrations with respect to ${\hbox {K}}^{+}$and in the presence of antioxidant molecules.

**Supplementary Information:**

The online version contains supplementary material available at 10.1140/epjp/s13360-022-02581-2.

## Introduction

### Ion potassium sensor and the G rich oligonucleotide sequences

Potassium ion sensors are intensively studied mainly due to the importance of the role potassium plays in fundamental biological processes, also in connection to pathological conditions [1–3]. Biosensors are of interest in ${\hbox {K}}^{+}$ measurements in biological solutions such as urine, blood or cell medium, *in vivo* measurements as well as to the assessment of extra- and intracellular ${\hbox {K}}^{+}$ concentration fluctuations, where a dynamic biosensor response is demanded. Along with conventional analytical techniques [4, 5] two biosensor categories have stimulated the efforts of many researchers. The first exploits the properties of oligonucleotides sequences, with particular reference to the G-rich sequences  [4, 6–13] whereas the other uses potassium binding proteins (pbp) as active elements [14]. The second type of biosensors give excellent results by fusing pbps with cyan and yellow fluorescent protein variants to create ${\hbox {K}}^{+}$ sensors based on Förster resonance energy transfer—(FRET), but their development and production are considerable more complex and expensive compared to the first type. This paper attempts to determine and elucidate the limits and capabilities of CD spectra to indicate the actual ${\hbox {K}}^{+}$ concentration in a variety of solutions. It is known that the presence of cations such as ${\hbox {K}}^{+}$ and ${\hbox {Na}}{+}$ and their concentration contributes to drive the PS2.M folding into the parallel and anti-parallel conformations but a survey of the actual quantitative response based on exclusively CD spectroscopy in a set of different solutions was, to our knowledge, still to be outlined.

### G-quadruplex formation

When Watson and Crick published the model of DNA A and B in 1953, the predominant structure was interpreted to stand as a double stranded right-handed helix carrying the basis where the genetic code information is stored. Over and above that model some non-conventional secondary structures such as G-quadruplex (G4) were identified [15, 16]. Gel formation of guanilyc acid at millimolar concentrations were known since the 19th Century but published by Bang only in 1910 and its structure was resolved 50 years later by Gellert and colleagues [17]. The crystal structure suddenly clarified the tetrameric association of guanines to form a G-quartet [17, 18]. The coupling of the 4 guanines forms a G-quartet or tetrad, which is the planar square structural unit with the guanines located at the four vertices [19, 20]. The base pairings are tied by Hoogsteen type H-bonds and each guanine becomes a bond donor and an acceptor as well. In particular, the pairing of the N1 on the first guanine with the O6 on the second guanine along with the pairing of N2 on the first guanine with the N7 on the second guanine result in eight hydrogen bonds per G-tetrad [15]. The G4 structure is obtained only when at least two tetrads pile up on each other. The thermodynamic stability is provided by different players, such as hydrophobic interactions ($\pi -\pi$ interactions) among the tetrads, and monovalent or divalent cations [19, 21]. It is crucial to point out that ${\hbox {K}}^{+}$ and ${\hbox {Na}}{+}$ play an outstanding role in the G4 thermodynamic stability, as X-ray structural analysis proves that such ions fit into the G4 complex. In particular ${\hbox {K}}^{+}$ coordinates the eight guanine carbonilic oxygens (O6) protruding into the central hydrophobic cavity. Because of its atomic radius, ${\hbox {Na}}{+}$ can coordinate only four oxygens. The role of the ${\hbox {Na}}{+}$ and ${\hbox {K}}^{+}$ ions is to minimize the electrostatic repulsion forces among the carbonilic oxygens and to stabilize the G4 structure promoting its folding. Since ${\hbox {K}}^{+}$ has a higher coordination power with respect to ${\hbox {Na}}{+}$, it displays a larger affinity that leads to more structural stability. Weaker stabilizers, compared to ${\hbox {K}}^{+}$ and ${\hbox {Na}}{+}$, are Mg$^{2+}$, Li$^{+}$ and Cs$^{+}$ [15, 22]. The G4 structure can be inspected by means of various techniques, such as X-ray crystallography, NMR, mass spectrometry, UV-VIS and circular dichroism (CD) spectroscopy. The last technique is a common approach to explore several aspects of the macromolecule secondary structures, G4s included. In particular, when the tetrads are piled up the DNA strand polarities contribute to determine the intensity and components of the CD spectra. CD allows the qualitative attributions of the spectra, defining three structural polymorphisms: parallel (positive peak at 264 nm, negative peak at 245 nm), antiparallel (positive peak at 295 nm, negative peak at 260 nm) and hybrid (positive peak at 295 nm, 260 nm, negative peak at 245 nm). Despite the fact that some exceptions to these features have been recognized, such as the interpretation of the shoulder 260 nm to 270 nm , the previous assignments are commonly accepted in case of G4 structures [23]. G-rich sequences are located in strategic regions of the genome and they play a central role such as gene expression regulation, structural and functional architecture of DNA and as important biological targets for cancer treatment [24]. Several G4 structures have been also studied as biosensors for detection of various analytes, such as metal ions in solutions having a wide range of complexity, organic molecules, nucleic acids, proteins and recently, pathogens including SARS-CoV-2  [8, 25].

### Technical aspects of G4-based biosensors application

G4 biosensors are often coupled to probes such as hemin, an anionic porphyrin known as a cofactor of proteins and enzymes like peroxidases. Once G4 has shaped up hemin binds to G4 to form a DNAzyme. In order to use DNAzyme for measuring the ${\hbox {K}}^{+}$ concentration one should also monitor the catalytic activity through an appropriate reaction mediated by substrates such as ABTS (2,2-azino-bis(3-ethylbenzthiazoline-6-sulphonic acid)) or luminol and ${\hbox {H}_{2}\hbox {O}_{2}}$. These molecules are oxidized (ABTS or luminol) and reduced (${\hbox {H}_{2}\hbox {O}_{2}}$) and the reaction is monitored by UV-VIS or chemiluminescence spectroscopy. The requested role of ${\hbox {K}}^{+}$ in the peroxidase activity is to induce the folding of secondary and tertiary structures of guanine-rich oligonucleotide sequences, such as an apoenzyme, capable to bind hemin and to perform like a peroxidase [6, 9, 26], but the stability and kinetics of PS2.M folding are affected by the binding of hemin (data not shown), which is a necessary component of the whole process. Since DNAzyme catalyzes the ABTS or Luminol oxidation by ${\hbox {H}_{2}\hbox {O}_{2}}$the presence of antioxidant molecules in the tested solutions interfere with DNAzyme activity reducing ${\hbox {H}_{2}\hbox {O}_{2}}$thus biasing the value of the ${\hbox {K}}^{+}$ concentration [9]. Thus the CD spectroscopy validation of PS2.M in a probeless experimental setup enables the adoption of such oligonucleotide as biosensor in chemical conditions where otherwise hemin would interfere with the PS2.M conformation stability. The absence of hemin permits to measure ${\hbox {K}}^{+}$ in the presence of oxidant or antioxidants that would disrupt the DNAzyme activity and avoids the adoption of substrates [27]. Other interesting potassium biosensors are based on G4-crystal violet complexes [13] where CV fluorescence variations are tied to the potassium concentration. These sensors are coupled to the CV molecule and also in this case interactions with oxidizing or reducing molecules cannot be ruled out thus increasing the system complexity.

### CD spectroscopy-based validation of the ${\hbox {K}}^{+}$ biosensor

In order to have a preliminary evaluation of the PS2.M folding induced by the presence of ${\hbox {K}}^{+}$, CD spectra of ${\hbox {K}}^{+}$ solutions at concentrations ranging between 0 mM and 10 mM and constant ${\hbox {Na}}{+}$ concentration of 80 mM were collected. Likewise spectra were collected also for solutions of DMEM and ${\hbox {K}}^{+}$ at the same concentrations. We then took spectra of solutions of $\hbox {KCl}$ and K:D-rib (a water solution of D-ribose and $\hbox {KHCO}_3$) separately for equivalent ${\hbox {K}}^{+}$ concentrations with the purpose of comparing the response observed in the CD spectra and rule out the possibility that K:D-rib could interfere with the PS2.M ${\hbox {K}}^{+}$ driven folding process. The adoption of a probeless sensor is crucial when measuring ${\hbox {K}}^{+}$ released by dissociation of substances such as K:D-rib which have antioxidant properties because they interact with the DNAzyme and its substrates (${\hbox {H}_{2}\hbox {O}_{2}}$and ABTS) thus demanding the interaction between them to be taken into account and thus adding complexity to the experimental data interpretation. In the last experiment PS2.M was tested in DMEM with K:D-rib. In this experiment the crucial point of the contribution to the overall ${\hbox {K}}^{+}$ concentration brought by DMEM and K:D-rib together with the solution complexity were taken into account.

## Materials and methods

### The oligonucleotide

The PS2.M (5’-GTGGGTAGGGCGGGTTGG-3’) single-stranded oligonucleotide was purchased from Tema Ricerca srl, Bologna, Italy. The lyophilized oligonucleotide was promptly reconstituted by adding TE buffer: Tris (10 mM) (Sigma-Aldrich) 7.5 pH and EDTA (0.1 mM) (Sigma-Aldrich) in order to obtain a stock concentration (100 $\upmu$M) and then stored at $-20^\circ$C. The DNA was quantified by UV/VIS spectroscopy at RT; the molar extinction coefficient is $\epsilon _{254}={191\, 085.2}{\hbox { M}^{-1}\hbox { cm}^{-1}}$. Before quantification, the DNA solution was heated at 95$^\circ$C for 5 min then cooled back at 0$^\circ$C (water and ice) for 10 min.

### Folding solutions

Each folding solution was prepared from a starting solution composed of PS2.M (10 $\mu$M) and EDTA (0.1 mM) then adding the solutions described in the following sections. The wanted ${\hbox {Na}}{+}$ and ${\hbox {K}}^{+}$ concentrations were obtained adding distilled water so as to reach the final volume of 400 $\mu$l. The DNA and the EDTA concentrations were in agreement with the protocol outlined by Paramasivan et al.  [28]. The folding reactions occurred at room temperature (RT), in the darkness and they took 24h to complete.

### Solutions with $\hbox {NaCl}$ and $\hbox {KCl}$

The simplest PS2.M folding process promoted by ${\hbox {K}}^{+}$ in the presence of ${\hbox {Na}}{+}$ at ${80}\hbox { mM}$ was measured in a series of 5 samples where the ${\hbox {K}}^{+}$ concentrations were 0, 2.5, 5, 7.5 and 10 mM. The ${\hbox {Na}}{+}$ source was $\hbox {NaCl}$ (Sigma-Aldrich) while those of ${\hbox {K}}^{+}$ was $\hbox {KCl}$ (Sigma-Aldrich). These samples were obtained in agreement with Sect. 2.2.

### Solutions with DMEM and $\hbox {KCl}$

Dulbecco’s Modified Eagle Medium (DMEM low glucose—Gibco) was the most complex solution tested. DMEM is a widely known cancer cell medium. When DMEM is supplemented with fetal bovine serum (FBS—Gibco), L-glutamine (L-Glut—Gibco) penicillin/streptomycin (pen/strep Gibco) is called “complete DMEM”. In this paper, unless explicitly declared, with DMEM we mean the complete form of the medium whose chemical and biological composition is a key factor to be taken into account. To mimic a human cell line culturing process, DMEM was supplemented with FBS (10%), L-Glut (1%) and pen/strep (1%). Since FBS itself is a source of ${\hbox {Na}}{+}$ and ${\hbox {K}}^{+}$ after the preparation their concentration in DMEM was ${170.8}\hbox { mM}$ and ${6.45}\hbox { mM}$, respectively. Measurements were carried out on samples of DMEM and $\hbox {KCl}$ at 0, 5, 7.5, 11.2, 15 mM. Due to the FBS contribution the solutions had actual ${\hbox {K}}^{+}$ concentrations of 6.45, 11.45, 13.95, 17.65 and 21.45 mM. For experimental reasons DMEM supplemented with $\hbox {KCl}$ were diluted 1:2 with respect to final volume (${400}\,{\upmu \hbox {l}}$) that was obtained adding distilled water (see Sect. 2.2). Thus in the measured solutions the ${\hbox {Na}}{+}$ final concentration was ${85.4}\hbox { mM}$ and the ${\hbox {K}}^{+}$ final concentrations were 3.23, 5.73, 6.98, 8.83, 10.73 mM.

### Comparison between K:D-rib and $\hbox {KCl}$

With the purpose of estimating the possible interference on the process brought about by the antioxidant action of K:D-rib (see Sect. 1.1) a folding solution of K:D-rib, which in its own right is a contributor of ${\hbox {K}}^{+}$, was measured to verify whether the PS2.M folding could be explained in terms of the overall ${\hbox {K}}^{+}$ only. A K:D-rib (${250}\hbox { mM}$) stock solution was prepared weighting of D-ribose (Sigma-Aldrich) (150 mg) and of ${\hbox {KHCO}}_{3}$(Prolabo) (300 mg) in distilled water (${4}\hbox { ml}$), corresponding to a molar ratio of 1:3 and gently mixing the solution waiting for the $\hbox {CO}_{2}$dissipation  [29]. To assay K:D-rib sample, the folding solution was prepared by adding K:D-rib stock solution and distilled water to the starting solution (see Sect. 2.2) to obtain K:D-rib at ${5}\hbox { mM}$. PS2.M folding in the presence of K:D-rib 5mM was compared with those in the presence of a sample carrying $\hbox {KCl}$ ${15}\hbox { mM}$, in agreement with what has been detailed in the Sect. 2.2 [10]. ${15}\hbox { mM}$ it is also the highest $\hbox {KCl}$ concentration used in the experiment with DMEM.

### Solutions with DMEM and K:D-rib

Lastly the CD spectra were measured for a batch of DMEM solutions supplemented with K:D-rib at 0, 1.5, 2.5, 3.5, 4.0, 5.0, 6.0,7.0, 10 and 12 mM. For experimental reasons such samples were diluted 1:2 with respect to final volume (${400}\,{\upmu \hbox {l}}$) that was obtained by adding distilled water (see Sect. 2.2). Considering the K:D-rib stoichiometry (Sect. 2.5) and the dilutions the ${\hbox {Na}}{+}$ concentration was ${85.4}\hbox { mM}$ and the final sample ${\hbox {K}}^{+}$ concentrations were: 3.22, 5.47, 6.97, 8.47, 9.23, 10.73, 12.23, 13.73, 18.23 and 21.23 mM.

### Circular dichroism

The CD spectra were collected by means of a J-715 spectropolarimeter (Jasco), endowed with an electronic Peltier to control the temperature of the cell holder. Spectra were measured in the 235 nm to 320 nm wavelength range at a scanning speed of 50 nm min$^{-1}$, data pitch of ${0.2}\hbox { nm}$, bandwidth of ${1}\hbox { nm}$ and time response of 8 s. Samples were held in a quartz cell with an optical path of 2 mm. The lamp holding chamber was flushed with a constant nitrogen stream to prevent ozone formation. All spectra were acquired at ${20}\,{^{\circ }}\hbox {C}$ and the final spectra were computed by averaging three subsequent spectra acquisitions. A reference spectrum was measured by acquiring a spectrum of a sample with TE instead of DNA and then subtracted from each sample spectrum.

## Results and discussion

### Solutions with $\hbox {NaCl}$ and $\hbox {KCl}$

**Fig. 1:**
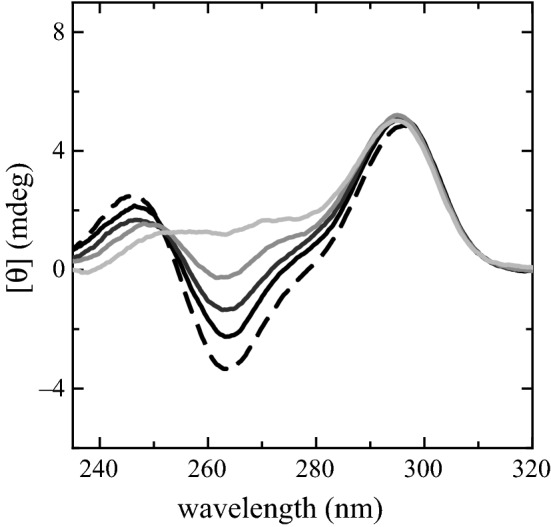
CD spectra of PS2.M (${10}\,{\upmu \hbox {M}}$) folded in the presence of the starting solution (distilled water and EDTA) supplemented with $\hbox {NaCl}$ (${80}\,{\upmu \hbox {M}}$) and $\hbox {KCl}$ at the concentrations of ${0}\,{\upmu \hbox {M}}$ (dashed black spectrum), ${2.5}\hbox { mM}$ (black solid spectrum), ${5}\hbox { mM}$ (dark gray solid spectrum), ${7.5}\hbox { mM}$ (gray solid spectrum) and ${10}\hbox { mM}$ $\hbox {KCl}$ (light gray spectrum). The experimental data were taken from our previous published paper [10]

**Fig. 2:**
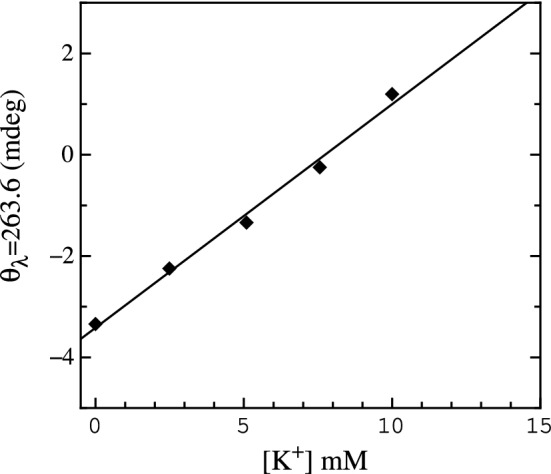
CD ellipticity values at ${263.6}\hbox { nm}$ as a function of the different ${\hbox {K}}^{+}$ concentrations in the presence of ${80}\hbox { mM}$ $\hbox {NaCl}$. The black solid line is the fitted linear regression.The experimental data were taken from our previous published paper [10]

We already described in [10] the PS2.M capability as ${\hbox {K}}^{+}$ biosensor in solutions of $\hbox {NaCl}$ and $\hbox {KCl}$. Examples of the spectra obtained in such experiment are shown in Fig. 1. These spectra have the typical spectral form characterizing antiparallel G4 structures as demonstrated also in [21, 28]. Such spectra display two positive peaks at ${~295}\hbox { nm}$ and ${~245}\hbox { nm}$, respectively, and a negative one at ${~265}\hbox { nm}$. Spectra amplitudes at ${~245}\hbox { nm}$ and ${~265}\hbox { nm}$ vary with the $\hbox {KCl}$ concentration. In particular, the component at ${~245}\hbox { nm}$ decreases and slightly shifts towards longer wavelengths while the component at ${~263}\hbox { nm}$ becomes less pronounced, almost vanishing at ${10}\hbox { mM}$ of $\hbox {KCl}$. The third component at ${~295}\hbox { nm}$ is nearly constant in intensity at all $\hbox {KCl}$ concentrations, beside a shift towards shorter wavelengths and a fourth positive shoulder at ${~275}\hbox { nm}$. The component at ${~275}\hbox { nm}$ shows up when ${\hbox {K}}^{+}$ is present in the solution getting more pronounced as the $\hbox {KCl}$ concentration grows. The largest ellipticity differences are observed at ${263.6}\hbox { nm}$. The ellipticity at ${263.6}\hbox { nm}$ in such spectra, plotted as a function of [${\hbox {K}}^{+}$] (Fig. 2), exhibits a linear dependence, whose linear fit is assumed to be the biosensor calibration curve. Figure 2 experimental data were taken from our previous published paper  [10] (Fig. 12, top panel, blue diamonds). In the reported potassium range the biosensor has a sensitivity of 0.44 ± 0.02 mdeg/mM  [10].

### Solutions with DMEM and $\hbox {KCl}$

**Fig. 3:**
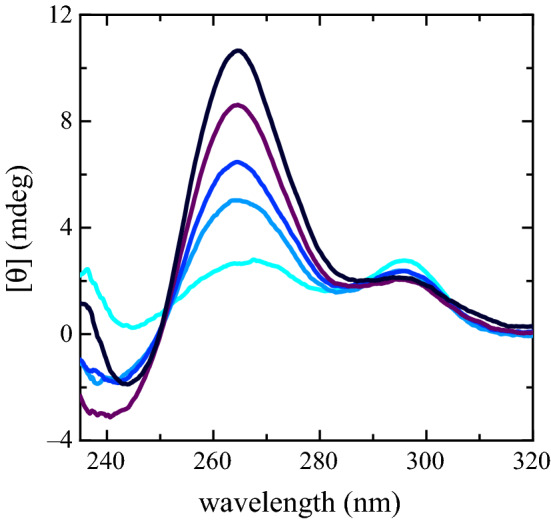
CD spectra of PS2.M (${10}\,{\upmu \hbox {M}}$) folded in the presence of the starting solution (distilled water and EDTA) supplemented with DMEM (turquoise spectrum) [${\hbox {Na}}{+}$] = ${85.4}\hbox { mM}$ and [${\hbox {K}}^{+}$] = ${3.23}\hbox { mM}$; DMEM treated with $\hbox {KCl}$ ${5}\hbox { mM}$ (light blue spectrum) [${\hbox {Na}}{+}$] = ${85.4}\hbox { mM}$ and [${\hbox {K}}^{+}$] = ${5.73}\hbox { mM}$; DMEM treated with $\hbox {KCl}$ ${7.5}\hbox { mM}$ mM (blue spectrum) [${\hbox {Na}}{+}$] = ${85.4}\hbox { mM}$ and [${\hbox {K}}^{+}$] = ${6.98}\hbox { mM}$; DMEM treated with $\hbox {KCl}$ ${11.2}\hbox { mM}$ (dark purple spectrum) [${\hbox {Na}}{+}$] = ${85.4}\hbox { mM}$ and [${\hbox {K}}^{+}$] = ${8.83}\hbox { mM}$; DMEM ${15}\hbox { mM}$ $\hbox {KCl}$ (black spectrum) [${\hbox {Na}}{+}$] = ${85.4}\hbox { mM}$ and [${\hbox {K}}^{+}$] = ${10.73}\hbox { mM}$

**Fig. 4:**
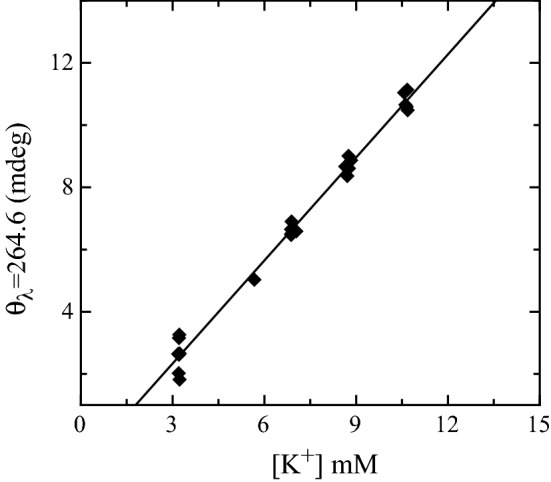
CD ellipticity values at ${264.6}\hbox { nm}$ as a function of the different ${\hbox {K}}^{+}$ concentrations coming from several PS2.M independent folding experiments in the presence of DMEM and DMEM treated with increasing $\hbox {KCl}$ concentrations. The black solid line is the fitted linear regression. For one run, the CD spectra corresponding to the whole ${\hbox {K}}^{+}$ concentrations investigated, are plotted in Fig. 3

Spectra of folded PS2.M in solutions of $\hbox {KCl}$ and DMEM are shown in Fig. 3. The components at ${~265}\hbox { nm}$ and ${~295}\hbox { nm}$ show the same kind of modifications of the $\hbox {NaCl}$–$\hbox {KCl}$ experiment, suggesting that PS2.M still folds into a G4 structure, despite the complexity of DMEM. The ellipticity signal magnitude at ${~295}\hbox { nm}$ weakly depends on the ${\hbox {K}}^{+}$ concentration and exhibits a small reduction corresponding to the growth of the component at ${~265}\hbox { nm}$. The wavelength where the CD spectra exhibit the largest variation is ${264.6}\hbox { nm}$. The wavelength ellipticity plotted as a function of ${\hbox {K}}^{+}$ has been adopted to characterize the biosensor response (Fig. 4) defining a resolution and a sensitivity of ${0.8}\hbox { mM}$ and 1.10±0.03 mdeg/mM, respectively.

**Fig. 5:**
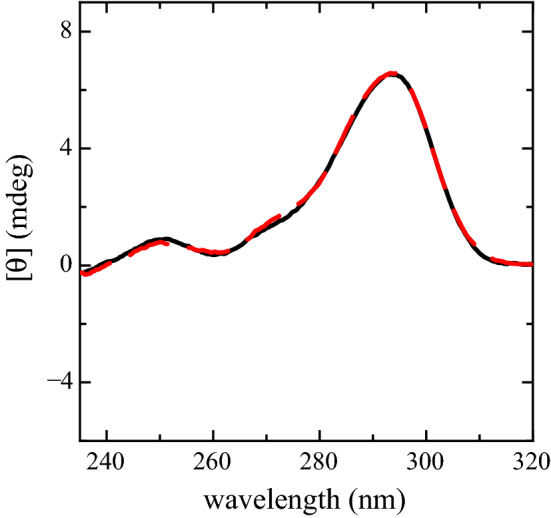
CD spectra of PS2.M (${10}\,{\upmu \hbox {M}}$) folded in the presence of the starting solution (distilled water and EDTA) supplemented with ${5}\hbox { mM}$ of K:D-rib (solid spectrum) and $\hbox {KCl}$ ${15}\hbox { mM}$ (red dashed spectrum)

### Comparison between K:D-rib and $\hbox {KCl}$

As explained in Sect. 2.5 we carried out these measures with the aim of understanding whether the presence of an antioxidant molecule (K:D-rib) somehow interferes with the folding of PS2.M, whether this substance could be considered a net contributor of ${\hbox {K}}^{+}$ to the solution and to what extent the spectra could be interpreted by the ${\hbox {K}}^{+}$ concentration. For this purpose two spectra were measured: the first of a PS2.M solution in the presence of K:D-rib at ${5}\hbox { mM}$, which means a ${\hbox {K}}^{+}$ concentrations of ${15}\hbox { mM}$, and the second in the presence of $\hbox {KCl}$ at ${15}\hbox { mM}$. The two corresponding spectra are shown together in Fig. 5. Both of them show the same intensity absorption at ${~295}\hbox { nm}$ (the highest peak), ${~250}\hbox { nm}$ (the lowest positive peak) and ${~272}\hbox { nm}$ (the positive shoulder), the component at ${~295}\hbox { nm}$ being characteristic of an antiparallel structure [26]. The positive shoulder at ${~272}\hbox { nm}$ becomes noticeable according to the spectra of the $\hbox {NaCl}$–$\hbox {KCl}$ experiment. In these experiments where both $\hbox {KCl}$ and K:D-rib [29, 30] were involved, CD spectra demonstrate the two ${\hbox {K}}^{+}$ sources induce PS2.M to fold according to the same G4 structure, despite the different chemical identities of K:D-rib and $\hbox {KCl}$; a similar behaviour was seen in other oligonucleotide sequences by Rehm and Colleagues [31] where identical spectra were also induced by different potassium salts, therefore not dependent on the anion.

**Fig. 6:**
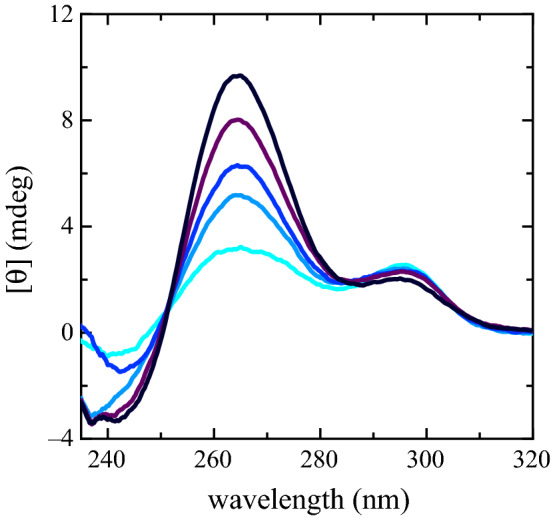
CD spectra of PS2.M (${10}\,{\upmu \hbox {M}}$) folded in the presence of the starting solution (distilled water and EDTA) supplemented with: DMEM (turquoise spectrum) [${\hbox {Na}}{+}$] = ${85.4}\hbox { mM}$ and [${\hbox {K}}^{+}$] = ${3.22}\hbox { mM}$; DMEM treated with K:D-rib ${1.5}\hbox { mM}$ (light blue spectrum [${\hbox {Na}}{+}$] = ${85.4}\hbox { mM}$ and [${\hbox {K}}^{+}$] = ${5.47}\hbox { mM}$; DMEM treated with K:D-rib ${2.5}\hbox { mM}$ (blue spectrum) [${\hbox {Na}}{+}$] = ${85.4}\hbox { mM}$ and [${\hbox {K}}^{+}$] = ${6.97}\hbox { mM}$; DMEM treated with K:D-rib ${3.5}\hbox { mM}$ (dark purple spectrum) [${\hbox {Na}}{+}$] = ${85.4}\hbox { mM}$ and [${\hbox {K}}^{+}$] = ${8.47}\hbox { mM}$; DMEM treated with K:D-rib ${5.0}\hbox { mM}$ (black spectrum) [${\hbox {Na}}{+}$] = ${85.4}\hbox { mM}$ and [${\hbox {K}}^{+}$] = ${10.73}\hbox { mM}$

### Solutions with DMEM and K:D-Rib

**Fig. 7:**
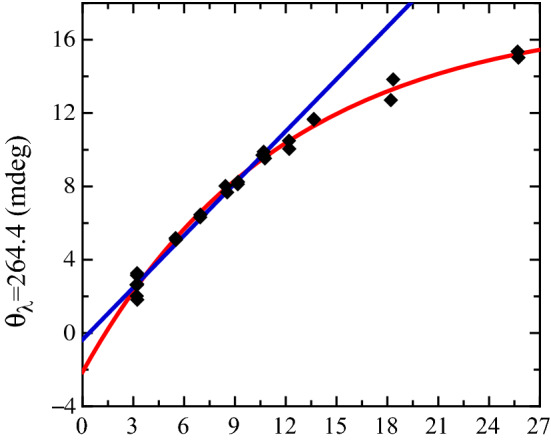
CD absorption values at ${264.4}\hbox { nm}$ as a function of the overall ${\hbox {K}}^{+}$ concentrations coming from DMEM and DMEM treated with K:D-rib. The plotted values are collected by several independent experiments of PS2.M folding. The lilac line is the fitted linear regression as if the biosensor would not have been saturated. The red one is the fitted exponential curve. They share the linear trend for [${\hbox {K}}^{+}$] ${<~11}\hbox { mM}$. CD spectra corresponding to the some of the ${\hbox {K}}^{+}$ concentrations investigated are plotted in Fig. 6

Finally, spectra generated by solutions of PS2.M folding in the presence of DMEM treated with increasing concentrations of K:D-rib (0 mM to 12 mM) were measured. These samples followed the same experimental procedure of the previous set, with K:D-rib playing the role of $\hbox {KCl}$. Also in this experiment the wavelength at which the CD spectra show the largest variations were observed at about ${264.4}\hbox { nm}$ as shown in Fig. 6, where a few selected spectra are displayed with the purpose of exemplify their behaviour. In this case too PS2.M folds according to a G4 structure. Even for this batch of samples, the CD spectra display the same two positive components at ${~265}\hbox { nm}$ and ${~295}\hbox { nm}$ with the component at ${~295}\hbox { nm}$ only weakly depending on the K:D-rib concentration. The presence of isodichroic points at ${~250}\hbox { nm}$ and ${~285}\hbox { nm}$ could be a sign of a two state transition from the antiparallel to parallel conformation similarly to what Zhang et al. observed in [12] for the PW17 oligonucleotide. No evident shift is present at ${~265}\hbox { nm}$ which displays a marked dependence on the K:D-rib concentration. Similarly to the other experiments, the absorption value at ${264.6}\hbox { nm}$ plotted as a function of the overall ${\hbox {K}}^{+}$ concentration is used to describe the response of the sensor, being the ${\hbox {K}}^{+}$ concentrations determined by contributions from both DMEM and K:D-rib. A calibration curve in the studied range can be obtained with an exponential fit, as shown in Fig. 7. The absorption spectrum changes over the whole K:D-rib concentration range but the linear response is best obtained for [${\hbox {K}}^{+}$] ${<11}\hbox { mM}$, leading to a resolution of ${~1}\hbox { mM}$ and sensitivity of 0.95 ± 0.03 mdeg/mM. All the spectra data and the datasets analysed during the current study are available as supplementary information to this paper.

## Conclusions

Even in a probeless experimental setup PS2.M reveals ${\hbox {K}}^{+}$ biosensor capabilities in water solutions in the presence of an excess of ${\hbox {Na}}{+}$. The sensitivity tests began with the simplest water solution composed by ${80}\hbox { mM}$ of $\hbox {NaCl}$ and an increase $\hbox {KCl}$ concentration. Resolution and sensitivity confirm the reliability of PS2.M as a ${\hbox {K}}^{+}$ biosensor in such conditions. The spectra suggest that K:D-rib works as a ${\hbox {K}}^{+}$ source through dissociation, since we observed the same spectra we had when the folding was promoted by adding $\hbox {KCl}$, which we take as a confirmation that PS2.M folds according to a G4 structure precisely in the same way. In addition, according to CD spectra D-ribose seems not to affect neither the PS2.M folding nor the ${\hbox {K}}^{+}$ availability. Next we tested the biosensor performance into a water solution with significant complexity such as DMEM treated with $\hbox {KCl}$ and K:D-rib. In both cases PS2.M displayed a linear response with respect to the ${\hbox {K}}^{+}$ concentration confirming the performance expectations, even at significantly higher ${\hbox {Na}}{+}$ concentrations.

This is certainly a very promising result. In fact other sequences studied to date such as PW17 [12] lose the linear response when in the presence of a high concentration of sodium like in extracellular matrices. The sensors presented in this paper and in [13] work in a similar way, attaining their linear behavior at similar potassium ranges and modulating the sodium concentration in order to obtain the optimal value in which the sensors work. Still in the conditions outlined the PS2.M sensor is based only on the G4 complex, while maintaining a linear response. Moreover it shows a much higher slope (1.10 ± 0.03 mdeg/mM) in the complex solution with respect to the $\hbox {NaCl}$–$\hbox {KCl}$ buffer solutions (0.44 ± 0.02 mdeg/mM), hence a higher sensitivity. This is not the case of the Crystal violet-G-quadruplex complexes tested on urine where the slope of the linear plot was not so large as that in the synthetic matrix [13]. It is important to emphasize how the strength of this sensor is precisely its ability to work at concentrations of ${\hbox {Na}}{+}$ useful for biological matrices and in the absence of other fluorescent probes.

The resolution is about the same (1 mM) in all the three calibration curves presented. The sensitivity are higher in the presence of DMEM treated with K:D-rib and $\hbox {KCl}$, with respect to the simplest solution, suggesting that the G4 dynamic, switching from antiparallel or mixed type conformations to parallel ones at defined potassium concentrations, depends on the solution compositions and complexity. This suggests that different calibration curves be made for each investigated solution in order to have the biosensor response correct readings. A similar experiment was performed using two non-synthetic oligonucleotides Pu27 and Pu23 (a Pu27 mutant) [11]. PS2.M displays a higher sensitivity and resolution respect to Pu27 and Pu23, that may be connected to their thermodynamic instability. Pu27 and Pu23 have relevant roles into the gene expression regulations, so it is not conceivable to have an unstable gene expression regulator. PS2.M could also work as a ${\hbox {K}}^{+}$ biosensor in biological matrices, such as cell supernatants, groundwater, marine or lacustrine water.

## Supplementary Information

Below is the link to the electronic supplementary material.

Supplementary file 1 (xlsx 35 KB)

Supplementary file 2 (xlsx 122 KB)

Supplementary file 3 (xlsx 35 KB)

Supplementary file 4 (xlsx 104 KB)

Supplementary file 5 (xlsx 22 KB)

Supplementary file 6 (xlsx 34 KB)
